# Modern Sociology

**Published:** 1902-08-02

**Authors:** 


### Modern Sociology.

### THE HONOURABLE ORDER OF SPINSTERS.

One is sometimes tempted to think that women—educated women, that is, who have entered the labour market—ask too much of their work. They ask not only that t should be honest and remunerative, but interesting and satisfying also. The ordinary man, on the contrary, appears to be quite happy if the two first requisites are supplied, and to find the interest and satisfaction of life in the domestic hearth. Miss Collet* thinks the explanation may be found in the lack of opportunity for exercising what may be called the maternal faculties; the woman, she says, is living an isolated life, and, unless her work involves the exercise of these faculties, an unnatural one. "With men, what they earn is of more importance than what they do." On the other hand, it is only those, both of men and women, who are genuinely and deeply interested in their work, who rise to any position of eminence in it, or who are likely to be highly paid, and she adds that "although high pay may compensate for uninteresting work, a woman will never be worth high pay if the work does not interest her. And we find, therefore, the paradoxical result that, generally speaking, the women who earn the highest incomes are the women who have chosen their work for the work's sake." An exception readily occurs in the nursing profession, but probably Miss Collet would hold that the nurses were themselves to blame for this, and that by resolutely holding out for adequate remuneration the state of things might be altered. A dearth in the supply, however, would be necessary before this could be effected.

It may be said that all work requiring the power of organisation gives opportunities for the development of the maternal instinct, taking the expression in its broadest sense, as we imagine Miss Collet uses it. Heads of institutions, colleges, etc., have great scope for its exercise, and, in a lesser degree, so have those who only rule a work-room or a class. The woman who inspires affection in her pupils is the one who is likely to get the best results, not the one who rules with a military despotism. Again, the medical profession, as exemplified by the women who stand at its head in our own country, develops the motherly faculties in a high degree.

May not gardening be said to meet the case, and does not the child who pulls up her plants "to see if they are growing" unconsciously exhibit the faculty in question? Work that may be drudgery to an untrained mind sometimes becomes a career to the trained practiser of it, and to the lover of nature for her own sake, the care of gardens offers not only a profession but a delight that every year may renew. "The miracle of spring, the snowdrop and crocus, the splendid pageant of summer, the treasure of harvest tide, and the bare fields but cosy hearths of Yule"—these things, as a modern poet says, are a sealed book to the uninitiated—to the person who loves the country only for its opportunities for sport—but they are an ever-increasing joy to the woman who has taken up gardening with the sincere purpose of making it her life-work, and finding in it scope for all her powers of brain and body. (Flippant people might be inclined to put the latter first, and to say with a popular paper, that the primary requisite for a gardener is a cast-iron back with a hinge in it; but gardening is not all spade-work, and the girl who goes through a course of training at a horticultural college will find that it is many-sided.) "Also," says the writer of one of the many garden books recently published, "gardening is the most cheerful and satisfactory pursuit for women who love outdoors."

The care of little children and helpless people, of course, is the most obvious occupation supplying the requisite quality, and at the opposite pole may be placed purely mechanical work in an office. "Clerical work in the case of the average woman," Miss Collet says, "can rarely be in itself satisfying; it is a means, not an end."

"Isn't it very dull and uninteresting?" we once asked a lady clerk in an office where only professional men's daughters are employed.

"It depends on whether you make it interesting," was the rather ambiguous reply; and we were left to infer either that the fileing and docketing of letters could be made interesting in itself, or that the opportunities of social intercourse which life in such an office might lead to, together with the regular recurrence of pay-day, compensated for much. The test question of course would be, "Would you do it if you were not paid to do it?" and the answer to this would probably be an emphatic negative.

Again, all artistic work—anything that produces a tangible result apart from the question of payment—must have a high place in our list of professions. The artist, whether she deals with colour, form, sound, or words, has a constant and growing sense of mastery of her tools, provided that she is honest and loves her work. Hannah More, Jane Austen, Maria Edgeworth, Caroline Herschell, Harriet Martineau, women of brilliant intellect, found their happiness in their work, and one does not hear that they were unhappy because unmarried. In literature, journalism, or scientific research, and in the human relationships that are the lot of all women who have hearts, they found their satisfaction and scope for the motherly instincts, and we can each think of women in our own circle of acquaintance who are satisfied in the work they have chosen, and who do not look upon marriage as the thing to be aimed at. "To marry, to settle, to grow stout, and at the last to be 'Jane, wife of the above, aged 74. Until the day break and the shadows flee away.' Unthinkable prospect!" says a modern writer, and it sums up, somewhat brutally, the unexpressed feeling of many women to-day.

It may safely be said that it is only when women are courageous enough to face a single life that they are likely to succeed as members of any profession; they will then be content to spend time and trouble in learning their work, and without special training a woman had better not enter the labour market at all. In this connection, Miss Collet inquires into women's prospects of marriage, and after minute statistics she arrives at the conclusion that "in all England and Wales the proportion of women who may be expected to remain unmarried is 1 in 6, in London it is 1 in 5." The figures, she says, have been called startling and alarming, but she fails to see why they should alarm anyone. "If all these spinsters had to be shut up in convents, the outlook would be gloomy. But as things are, if only we can secure good pay and decent conditions of life, the lot of all women may be immensely improved by this compact band of single women. It would be difficult to overrate the industrial effect of a number of well-instructed, healthy-minded, vigorous permanent spinsters. A man's work is not interrupted but rather intensified by marriage; but in the case of women, not only is the wages question very much affected by the expectation of marriage, but much organised effort on their part, whether for improvement of wages or for provision against sickness and old age, must be wasted unless there be a considerable number of single women to give continuity to the management of their associations . . ." and she professes a faith in the absolute necessity for single women which is reassuring.

* "Educated Working Women." P. S. King. 1902.

